# Fatal cardiotoxicity related to halofantrine: a review based on a worldwide safety data base

**DOI:** 10.1186/1475-2875-8-289

**Published:** 2009-12-10

**Authors:** Olivier Bouchaud, Patrick Imbert, Jean Etienne Touze, Alex NO Dodoo, Martin Danis, Fabrice Legros

**Affiliations:** 1 Service des maladies infectieuses et tropicales, Hôpital Avicenne AP-HP et Université Paris 13, 125 rue de Stalingrad, 93009 Bobigny, France; 2 Service des maladies infectieuses et tropicales, Hôpital d'Instruction des Armées Bégin, 69 avenue de Paris, 94160 Saint-Mandé, France; 3 Ecole de Santé des Armées du Val de Grâce, Place A. Laveran, Paris 75230, France; 4 Centre for Tropical Clinical Pharmacology & Therapeutics, University of Ghana, Medical School Korle-Bu Teaching Hospital, Accra, Ghana; 5 Service de Parasitologie Mycologie, Centre National de Référence du Paludisme, CHU Pitié-Salpêtrière, 47-83 boulevard de l Hôpital, 75651 Paris cedex 13, France; 6 French Malaria National Reference Centre, Paris, France

## Abstract

**Background:**

Halofantrine (HF) was considered an effective and safe treatment for multi-drug resistant falciparum malaria until 1993, when the first case of drug-associated death was reported. Since then, numerous studies have confirmed cardiac arrythmias, possibly fatal, in both adults and children. The aim of the study was to review fatal HF related cardiotoxicity.

**Methods:**

In addition, to a systematic review of the literature, the authors have had access to the global safety database on possible HF related cardiotoxicity provided by GlaxoSmithKline.

**Results:**

Thirty-five cases of fatal cardiotoxicity related to HF, including five children, were identified. Females (70%) and patients from developing countries (71%) were over-represented in this series. Seventy-four percent of the fatal events occurred within 24 hours of initial exposure to HF. Twenty six patients (74%) had at least one predisposing factor for severe cardiotoxicity, e.g., underlying cardiac disease, higher than recommended doses, or presence of a concomitant QT-lengthening drug. All (100%) of the paediatric cases had either a contraindication to HF or an improper dose was given. In six cases there was no malaria.

**Conclusion:**

A distinction should be made between common but asymptomatic QT-interval prolongation and the much less common ventricular arrhythmias, such as torsades de pointes, which can be fatal and seem to occur in a very limited number of patients. The majority of reported cardiac events occurred either in patients with predisposing factors or with an improper dose.

Therefore, in the rare situations in which HF is the only therapeutic option, it can still be given after carefully checking for contraindications, such as underlying cardiac disease, bradycardia, metabolic disorders, personal or family history of long QT-interval or concomitant use of another QT-prolonging drug (e.g., mefloquine), especially in females.

## Background

Halofantrine (HF), a phenanthrenemethanol derivative of aminoalcohol, was first marketed in 1988. It was considered effective and safe for treating malaria, including multidrug resistant *P falciparum *strains [1] until 1993, when ter Kuile *et al *reported the first death related to HF cardiotoxicity [2]. Of note is that HF was given at higher doses than recommended (72 versus 25 mg/kg) to increase efficacy on the Thai-Burma border, an area with high level resistance [2]. This report led to a prospective electrocardiographic study which consistently showed HF dose related lengthening of the PR and QT intervals [3]. Since then numerous studies have confirmed cardiac toxicity in both adults and children, and several cases of death or severe cardiac toxicity of HF have been reported [4-15]. Due to these potentially severe side effects, and despite a reliable and rapid efficacy, usage of HF decreased dramatically in both malaria-endemic and industrialized countries.

Since the majority of reported cardiac accidents appeared to occur either in patients with predisposing factors or at higher than recommended doses, the aim of the study was to collect and carefully examine all reported fatal cases of HF-related cardiotoxicity [15-18]. While there are currently safer and equally effective anti-malarial drugs available (e.g., artemisinin derivatives, atovaquone-proguanil), the prospect of new drugs becoming available over the next decade is limited due to financial constraints. Thus, it seems reasonable to carefully examine the risk/benefit ratio of HF and either discard it altogether, or better define that ratio for possible use should the need for it arise, e.g. the occurrence of resistance or unexpected side-effects of current anti-malarials.

## Methods

In March 2005, the Regulatory Affairs Department of GlaxoSmithKline (GSK)

(GlaxoSmithKline Research & Development Limited, New Frontiers Sciences Park, Harlow, Essex, UK) provided all mortality data of cardiac origin related to HF from the GSK Worldwide Global Clinical Safety and Pharmacovigilance Data base [19]. All spontaneous, post-marketing surveillance and clinical trial serious adverse events reports of fatal cardiac arrhythmia or death, where HF was reported as the suspect drug, were considered for identification of cases. To provide as comprehensive a report as possible, relevant key words were used including the followings: arrhythmia, atrioventricular block, bradycardia, bundle branch block, cardiac arrest, cardiac failure, cardio-respiratory arrest, cardiovascular disorder, chest pain, circulatory collapse, death and sudden death, electrocardiogram abnormal or change, extrasystoles, hypotension, long QT syndrome, myocardial infarction, palpitations, QT prolonged, shock, tachycardia, torsade de pointes, ventricular hypertrophy, ventricular fibrillation, ventricular tachycardia.

Reports were considered as unassessable when the details of neither the event nor the patient were available. In addition, reports were excluded when there was an alternative diagnosis much more likely to have caused the death. In addition to this systematic review of all cases of fatal cardiotoxicity possibly related to HF reported to the GSK database, all similar cases reported in the literature were reviewed. Doubles in both sources were identified by comparing available data (date, age, sex, country, case description, outcome).

## Results

**Figure 1:**
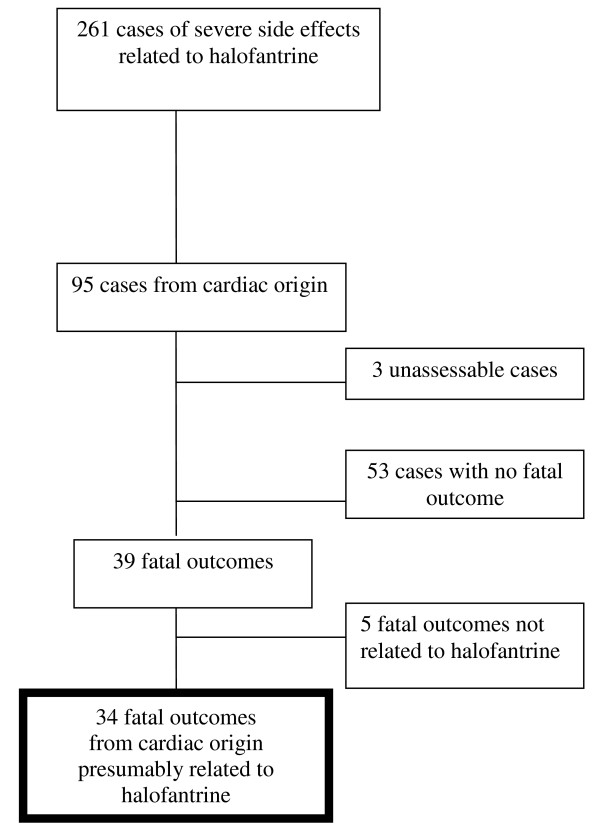
Selection of 34 cases* of fatal outcome from cardiac origin presumably related to halofantrine from the GSK Global Clinical Pharmacovigilance Data Base [18]. * an additional case not reported in the GSK data base was found in the literature.

GSK has estimated that 17.9 million patients, mainly in developing countries, were treated with HF between 1988 and 2005 (19). Of the 261 reports of severe HF related side effects reported to the GSK safety database, 95 had a cardiac explanation [19]. They were mainly reported from France (41%), Pakistan (14.7%), Germany (8.4%), and Kenya (6.3%). Fifty six cases were excluded because the only information available was exposure to HF and death in three cases and because of non-fatal outcome in 53 cases. Among the 39 remaining fatal cases, five were much more likely due to an unrelated cause (details not shown, available on demand), leaving 34 cases of cardiac fatalities suspected to be related to HF (Figure 1). An additional case published in the literature but not included in the GSK data base was identified, resulting in a total number of fatal cases of 35 [3].

Table 1 summarizes the main characteristics of these 35 patients and Table 2 displays the cumulative effect in patients having more than one predisposing factors. In 26 patients (74%) at least one predisposing cardiac factor (and up to three) was recorded including: concomitant medication with possible cardiac effect (n = 20), underlying cardiovascular disease or deleterious medical condition (n = 14), improper administration of HF (excess, n = 3, or drug taken with food, n = 2). One patient died from a cardiac arrest following a seizure.

**Table 1:** main characteristics of 35 cases of fatal outcome from cardiac origin presumably related to halofantrine

patient age^a ^(year)	Median: 27; range: 2 - 53
sex^b^	male: n = 9 (27%); female: n = 24 (70%)

geographic origin of patients	developing world, n = 25; developed world, n = 10

type of report	• spontaneous n = 34
	• clinical trial n = 0
(GSK data base only)	• post marketing survey n = 0

source of report	health care professional n = 35

time from first dose to death (day)	median: 1; range: 0 3
	
	• same day as first dose: n = 13 (37%)
	• 1 day after first dose: n = 13 (37%)
	• 2 days after first dose: n = 2 (6%)
	• 3 days after first dose: n = 3 (8%)
	• unknown: n = 4 (11%)

number of patients receiving 1 or 2 courses of halofantrive and number of doses taken by patients^c^	• first course of halofantrine: n = 23
	◦ 1 dose: n = 1 (3%)
	◦ 2 doses: n = 9 (26%)
	◦ 3 doses: n = 10 (28%)
	◦ other including pediatric formulation: n = 3
	• second course of halofantrine: n = 6
	◦ 5 or 6 doses: n = 5 (14%)
	◦ 8 doses: n = 1 (3%)

malaria diagnosis and malaria	• no diagnostic test performed: n = 8
species^d^	• blood smear negative: n = 7
	• *P. falciparum*: n = 8
	• *P. vivax*: n = 1
	• *Plasmodium sp*: n = 4

concomitant drugs with possible cardiac effect	n = 20 (57%)
	
	• anti-malarial: chloroquine: n = 7; mefloquine: n = 4; amodiaquine: n = 1;
	• antibiotics: cyclines, n = 2; metronidazole, n = 1; ciprofloxacine, n = 1; norfloxacine, n = 1
	• drugs leading to electrolyte imbalance: diuretics, n = 2; potassium, n = 1

underlying medical condition	n = 14 (40%)
	
	• cardiovascular disease, n = 11
	• obesity, n = 1
	• epilepsy, n = 1
	• severe anaemia, n = 1

missing data: a: n = 3; b: n = 2; c: 6; d: n = 7

**Table 2:** Classification of 35 patients with fatal outcome from cardiac origin presumably related to halofantrine (HF) according to the number of predisposing factors to cardiac complication and indication for halofantrine

Number of predisposing factor(s), number of patients (number of children)	Details on predisposing factors (underlying cardiac disease, concomitant medication, misuse of HF, etc)	Wrong or debatable indication for HF
no predisposing factors, n = 9 (0)	Pt # 2, 11§ 12, 16, 17, 18, 20, 31, 33	a* = 1, b* = 2, c* = 1, d* = 2

1 predisposing factor, n = 16 (2)	Pt # 1, 30: vibramycine	b* = 3, c* = 2
	Pt # 5, 8, 14, 19: chloroquine	e* = 1
	Pt # 7: bundle branch block¤	
	Pt # 9, 34: HF taken with food	
	Pt # 13: metronidazole	
	Pt # 21: potassium infusion	
	Pt # 23: unknown severe cardiac valve disease	
	Pt # 24: angina pectoris	
	Pt # 25: ciprofloxacine	
	Pt # 29: mefloquine	
	Pt # 32: HF overdose (45 mg/kg)	

2 predisposing factors, n = 5 (2)	Pt # 3: cardiomyopathy¤ + furosemide	a* = 1; c* = 1
	Pt # 4: obesity + chloroquine	d* = 1; f* = 1
	Pt # 6: tachycardia + mefloquine	
	Pt # 10: severe anaemia and dehydration + amodiaquine	
	Pt # 26: unknown right ventricular arrhythmogenic dysplasia + chloroquine	

3 predisposing factors, n = 5 (1)	Pt # 15: cardiac arrhythmia + chloroquine + amodiaquine	b*= 2; c* = 2
	Pt # 22: arteriosclerosis and high blood pressure + indapamide + HF taken with food	
	Pt # 27: unknown cardiomyopathy + mefloquine + erythromycin	
	Pt # 28: unknown right ventricular arrhythmogenic dysplasia + chloroquine + probable HF overdose°	
	Pt # 35: tachyarrhythmia¤ + mefloquine + HF overdose (72 mg/kg)	

*: a = no certainty of malaria diagnosis/no blood smear performed (industrialised country);

b = no certainty of malaria diagnosis/no blood smear performed (developing country);

c = no malaria (blood smear negative); d = possible severe malaria; e = severe malaria;

f = *P. vivax*

¤: known prior to HF prescription; §actual exposure to HF unsure; ° high HF blood level (see text)

Autopsies were performed in eight cases including three cases without an apparent cause of death appeared (in one of these three cases, brain pathology findings suggested cerebral malaria). In five cases cardiac abnormalities were noted, including one case where HF was cited as responsible in a 27 year-old Kenyan patient who had an history of cardiac arrhythmia and had received mefloquine for an acute malaria shortly before HF. For the four other patients pathologic findings were respectively, previously unknown right ventricular arrhythmogenic dysplasia (two young French females); hypertrophic cardiomyopathy in a 22 year-old patient from Togo (having taken mefloquine before HF); and a flabby myocardium and signs of multi-organ failure (congested kidneys, pulmonary oedema and congestion, and enlarged liver) in a 31 year-old female from Ghana.

Halofantrine levels measured by HPLC were available in three cases: two considered in the normal therapeutic range for both halofantrine and N-desbutyl-halofantrine [20] and one with high values (respectively 879 and 510 ng/mL) supporting a possible overdose [15,19]. Five cases occurred in children less than 16 y (three males and two females), living in a developing country (four) or in France (one). Mean age was 9.6 y (range: 2-13). All had a contraindication to halofantrine (including one case of cerebral malaria) or a concomitant medication, which could have been a predisposing factor for cardiac arrythmia (Table 3).

**Table 3:** Contraindication (and complicating drugs) to halofantrine in 5 children with fatal outcome of cardiac origin presumably related to halofantrine

patient #3 (13 y. old)	known history of cardiomyopathy (treated by furosemide)
patient #10 (2 y. old)	severe anaemia, dehydration (+ amodiaquine prior to halofantrine)

patient #15 (11 y. old)	known history of cardiopathy with cardiac arrhythmia (+chloroquine and amodiaquine prior to halofantrine)

patient #21 (8 y. old)	severe/cerebral malaria + bradycardia (+ potassium injection)

patient #32 (12 y. old)	HF overdosing (45 mg/kg) (+ concomitant norfloxacine + possible intake of amodiaquine)

Of the 35 cases, twenty-one (including one paediatric case) have been published either as case reports or included in a review published by Wesche *et al *from the FDA Spontaneous Reporting System (containing fewer details than those available in the GSK safety data base) [3,7,9,13,15,18,21].

## Discussion

Based on the analysis of the GSK Worldwide Global Clinical Safety and Pharmacovigilance data base and a review of the literature, it appears that 35 fatalities presumably related to HF, including five children, were reported from 1988, when the drug first became available, to March 2005. No additional cases have been published since. No cases were reported in clinical trials prior to marketing nor during the post-marketing phase. Besides possible under-reporting, another limitation is that reported cases often lack essential information such as the exact dosage, body weights, time course for development of arrhythmia or death, description of concomitant morbidity (especially heart diseases), usage of potentially complicating medications, electrocardiographic description, etc. With such insufficient data, there are two main problems with attempting to delineate cardiac risk with HF: first, other causes or predilecting factors, including those related to the malaria itself, may have not been reported. Indeed it appears that HF was incorrectly given in at least three cases of severe malaria (a case of clinical cerebral malaria and two other cases of autopsy-proven cerebral malaria with multi-organ failure). Second, in 15% of cases of sudden cardiac death in the general population, no cause is found despite extensive study, including autopsies [22,23].

Cardiac toxicity of HF was first reported in 1993 when a Thai woman with a life-long history of tachyarrhythmias died suddenly. She had received high-dose HF (72 mg/kg, *i.e *approximately three times the standard dose recommended) for a recrudescent falciparum malaria after failure of a mefloquine treatment [2]. As pre-marketing and early post-marketing safety studies had not shown previously any HF cardiac effect, electrocardiograms were not included in the high-dose halofantrine prospective study performed to validate the 72 mg/kg regimen in this area one year before that death [2]. A subsequent prospective electrocardiographic study performed by the same team showed that high-dose HF was associated with QTc interval prolongation [3]. The lengthening was correlated to HF and its metabolite plasma concentrations as reported in another study [3,24]. The magnitude of QTc prolongation was greater in patients whose HF treatment closely followed mefloquine. Since then several reports of HF-induced QTc prolongation, as well as severe ventricular arrhythmias and torsades de pointes, particularly in patients with congenital prolonged QT syndromes have been published [8-10,12,14,18,24,25]. In 1993, the discovery of electrocardiographic and possible clinical cardiac effects induced by HF has led the WHO and GSK, which produces HF, to recommend limitations on the prescribing of HF [21].

Assuming there is no reason for an over-representation of females receiving HF, it appears from the data shown here that females (70%) seem inordinately sensitive to fatal cardiac toxicity. There are, unfortunately, not enough available data to enable an analysis of weight as a possible risk factor of HF overdosing due to significant lower body weight in females. Within the general population it is known that QT interval is longer and QT prolongation and torsades de pointes are more common among females, who are more susceptible to torsades de pointes from QT-prolonging drugs [26,27]. However, there is no evidence for a greater risk of sudden death. On the contrary, the risk for females seems lower globally [26,27].

Twenty-five cases (71%) occurred in patients living in developing countries. This is not surprising as the denominator (malaria patients exposed to HF) is much higher (200 fold) compared to industrialized countries [19]. In addition, due to field conditions it is likely that side-effects were underreported in contrast to the higher rate of predisposing cardiac factors e.g., vitamin B1 deficiency (inducing QT prolongation) due to nutritional insufficiency, electrolyte abnormalities or cardiac rheumatism [28]. Thus, HF toxicity may have been underestimated in tropical areas.

Indeed 74% of patients had at least one predisposing cardiac factor and in five cases each, two and three factors (Table 2). Such pre-existing or concomitant predisposing factors have been highlighted by some authors to increase the HF-cardio-toxicity and to contribute to the onset of severe clinical manifestations of HF cardio-toxicity, HF-induced QT prolongation being the initial step [2,15,18]. In addition, the role of malaria itself cannot be excluded even if cardiac impairment in malaria is debated and unlikely in non-severe forms. However, HF could exacerbate malaria-associated orthostatic hypotension, making it a potentially predisposing factor [3,29,30]. While acknowledging the limits of available data in this study, it is interesting that no predisposing factors were observed in nine cases (26%). Among these nine cases, the diagnosis of malaria was unproven in 3 cases (including one case in a developed country), disproven in one case, and according to available data, severe malaria was likely in 3 cases, which constituted a contraindication to the use of HF. Of the 26 cases where at least one predisposing factor was identified, the diagnosis of malaria was unproven in six cases (including one in a developed country) and disproven in five cases (Table 2).

Death occurred after two or three doses in 65% of documented cases and within the first twenty four hours following first dose of HF in 74% of the cases. This is consistent with pharmacokinetic data on HF including the fact that QT-prolongation, which reaches a maximum 12 hours after first dose, is related to the plasma concentration of the parent drug which appears, according to Wesche *et al*, more cardiotoxic than its N-desbutyl metabolite [1,3,18,24].

The fatal event occurred during the first course of HF in 79% of the cases, assuming that, given the quality of data, the event occurred after the first course unless explicitly stated to have occurred during the second. This is not surprising as a second course was only recommended in non-immune patients, who represented only a small part of the population exposed to HF. However, electrocardiographic changes have been reported as greater during the second course than the first due to higher HF plasma concentrations, leading to a probable higher risk of severe outcome [24].

Paediatric HF cardiotoxicity has been documented, in the form of asymptomatic electrocardiographic changes, in almost half of treated children [10,14]. However the risk of severe cardiac complications was considered very low as, none of the numerous studies of HF when standard recommendations were followed, have shown symptomatic cardiac manifestations in either developing and industrialized countries [10,11,14,31-39]. In contrast, five fatal paediatric cases are reported in this study, all of which occurring in a context of contraindication or misuse of HF (Table 3). In addition, HF was given in one case of vivax malaria and in another case (in a European country) without proven malaria [19].

Statements about the danger of using HF may be unwarranted if they fail to discriminate between the frequent (as for other common drugs) but asymptomatic electrocardiographic changes limited to QT-interval prolongation and potentially fatal ventricular arrhythmias or torsades de pointes which occur in a very limited number of patients with probable predisposing factors after a first step of QT-prolongation [18,40,41]. HF has a high pro-arrhythmic potency, inducing the same concentration-dependant effects on QTc dispersion and variability as class-III anti-arrhythmic agents, which are known for their pro-arrhythmic action [18,30,42]. This pro-arrhythmic effect is due largely to the fact that HF, like mefloquine and lumefantrine, acts upon cardiac myocytes to block the potassium channels which initiate repolarization and determine the QT interval under genetic regulation (LQT1 and HERG genes) [16,17]. Although research in this area is relatively new, it may well be as has been recently postulated by Kannankeril that the ability for QT-prolonging drugs to induce ventricular arrhythmias or torsades de pointes is genetically determined [43,44]. An underlying cardiac impairment would seem to be an additional risk factor for a severe outcome [3,15]. Thus discerning how much of the problem is due to the inherent properties of HF can be difficult when there are multiple complicating factors. An excellent example was the first reported death related to HF, who was a female with a known pre-existing tachyarrhythmia, given an excessive dose of HF, and who had also recently taken mefloquine, a long half-life QT-prolonging drug [2].

The 53 non-fatal cases of HF associated cardiotoxicity in the sampled patients were not analysed as the data sources on cardiotoxicity were variable and as several other studies and methodologies have yielded robust information on HF associated cardiotoxicity. This study therefore focused exclusively on fatal cases of HF cardiotoxicity despite the obvious heterogeneity of the data sources and the challenges that posed to the review and analysis.

This review of fatal HF cardiotoxicity focused on 35 cases from the GSK Worldwide Global Clinical Safety and Pharmacovigilance data base, plus a review of the literature. This figure is very likely underestimated given that the vast majority of HF treatments took place in the developing world where pharmacovigilance activities are absent or newly developed and where adverse events to all medicines are poorly documented. It would be useful to compare this with fatalities due to comparable drugs such as mefloquine, but at the time of this review we were unable to access the safety data base of Roche laboratories. In contrast, no cardiac effects or deaths have been reported to date following treatment using lumefantrine, a parent drug of HF [30,45]. These major differences in cardiotoxicity between HF and lumefantrine, which are very similar drugs, are consistent with the known lesser cardiotoxicity of HF racemic compound and/or the HF N-desbutyl metabolite compared to HF itself [18].

HF is still used in countries such as Pakistan or parts of West and Central Africa, despite the availability of much safer options including the WHO recommended artemisinin combination therapies. In these countries, physicians should carefully check for contraindications, especially in females, such as any underlying heart disease, bradycardia, personal or family history of long QT-interval, concomitant use of another QT-prolonging drug (especially mefloquine) and only prescribe HF at the recommended dosage (25 mg/kg) to be taken on an empty stomach. In addition, HF should be strictly restricted to non-severe *P. falciparum *malaria in patients with no risk of severe electrolytes imbalance. Following these guidelines would greatly lessen the risk of cardiac side-effects [43]. In industrialized countries, where other safer and efficient anti-malarial agents are available, there is currently no real need for HF apart from unusual cases.

## Conclusion

The undoubted cardiotoxicity of HF should lead physicians to use safer drugs when available. Nevertheless, when there is no other choice, serious adverse outcomes could be significantly decreased if the drug were used according to current manufacturer recommendations. In addition, HF may again be needed if the limited number of new anti-malarial agents proves inadequate for future needs. In that case, the development of the N-desbutyl-metabolite or a racemic compound of HF would be preferable, as they seem significantly less cardiotoxic than the parent drug [18]. The development of pharmaco-genetic tools would possibly allow a safer use of HF and other QT-prolonging drugs by screening patients at particular risk for severe complications. Routine genotyping of patients appears unrealistic for the foreseeable future [43].

## Competing interests

The authors declare that they have no competing interests.

## Authors' contributions

OB participated in the design of the study, performed the analysis of data, wrote and reviewed the manuscript. PI participated in the design of the study, contributed to the analysis of data and reviewed the manuscript. JET participated in the design of the study and reviewed the manuscript. ANOD reviewed the manuscript. MD participated in the design of the study and reviewed the manuscript. FL participated in the design of the study and was in charge of contacts with the Regulatory Affairs Department of the GlaxoSmithKline Laboratory. All authors read and approved the final manuscript.
